# A New Classification System to Determine Posterior Mandible Morphology for Implant Therapy

**DOI:** 10.1155/ijod/3244223

**Published:** 2026-02-01

**Authors:** Faisal Alqaood, Jahanzeb Chaudhry, Amar Hassan Khamis, Keyvan Moharamzadeh, Moosa Abdulla Abuzayeda

**Affiliations:** ^1^ Al Jahra Specialty Dental Center, Al Jahra, Kuwait; ^2^ Department of Oral Diagnostics and Surgical Sciences, Hamdan Bin Mohammed College of Dental Medicine, Mohammed Bin Rashid University of Medicine and Health Sciences, Dubai, UAE, mbruniversity.ac.ae; ^3^ Hamdan Bin Mohammed College of Dental Medicine, Mohammed Bin Rashid University of Medicine and Health Sciences, Dubai, UAE, mbruniversity.ac.ae; ^4^ Department of Restorative Dentistry, Hamdan Bin Mohammed College of Dental Medicine, Mohammed Bin Rashid University of Medicine and Health Sciences, Dubai, UAE, mbruniversity.ac.ae

**Keywords:** alveolar ridge morphology, dental implants, inferior alveolar canal, lingual concavity, lingual undercut, mandible

## Abstract

**Background:**

We studied the posterior mandibular morphology to develop a new alveolar ridge morphology classification system for implant therapy. Current classification systems do not adequately address essential parameters such as the influence of lingual undercut on implant placement. They do not fully consider clinically important factors such as the shape of the alveolar ridge, the depth and angle of the lingual concavity, and the position of the inferior alveolar canal (IAC). This study was based on an existing classification system and culminated in the development of a new CPD classification system, with a primary focus on lingual concavity.

**Material and Methods:**

One hundred and ninety‐five patients aged above 19 to over 70 years were included in this cross‐sectional study. Cone‐beam computed tomography (CBCT) volumes of 90 males and 105 females were analyzed to determine the width of the alveolar bone at the crest (Wc), width of the alveolar bone at the base (Wb), alveolar ridge height (Vcb), alveolar bone height below the Point P (Vb), alveolar bone height above the Point P (Vc), and lingual concavity depth and angle. These parameters were selected to determine the ridge shape and the presence/absence of an undercut. These values were also analyzed in relation to age, gender, presence/absence of the first molar, and presence/absence of the lingual concavity.

**Results:**

The U‐type (undercut) ridge was the most common (54.4% left; 52.1% right), followed by the P‐type (parallel; 27.2% left; 33.5% right) and the C‐type (convergent; 18.5% left; 14.4% right). Alveolar ridge height was correlated with age, gender, ridge type, presence of the first molar, and presence of an undercut. The width of the alveolar bone at the crest correlated with the presence of the first molar and ridge type. The lingual concavity depth correlated with the presence of the first molar, undercut, and ridge type.

**Conclusions:**

We have developed and validated a new and comprehensive CPDU classification system for assessing the posterior mandible to enhance preoperative assessment for implant therapy by categorizing the alveolar ridge morphology into C‐type, P‐type, and D‐type with or without undercut (U) based on the ridge shape, lingual concavity depth and angle, and IAC position.

## 1. Introduction

Dental implants are routinely used to replace missing teeth with high success and survival rates [1]. Advancements in implantology enable a smooth implant placement process with pleasing esthetics and high patient acceptance. However, to ensure successful implant placement, proper diagnosis, treatment planning, and a thorough assessment of the implant site are necessary [2]. The assessment includes factors such as the height and width of the alveolar ridge, the position of the planned prosthesis, bone density, and the presence or absence of a lingual undercut [3, 4]. Careful assessment of the related anatomical structures, such as the nasal floor, maxillary sinus, incisive canal, mental foramen, and inferior alveolar canal (IAC), is essential to avoid complications [5].

The posterior region of the mandible contains vital structures such as the inferior alveolar nerve. When the nerve is injured, the patient may experience paresthesia and loss of sensation in the lower lip and chin [6–8]. The submental and sublingual arteries are in close proximity to the lingual cortical plate of the posterior mandible. Injury to the arteries during implant placement could cause hemorrhage and hematoma formation, which may lead to airway obstruction and post‐surgical infection [8–13]. Therefore, placing an implant in the posterior mandible requires a thorough assessment of the alveolar ridge morphology to determine the optimal implant insertion pathway that accommodates the lingual concavity (lingual undercut) and mandibular body angulation to avoid lingual cortical plate perforation.

**Figure 1:**
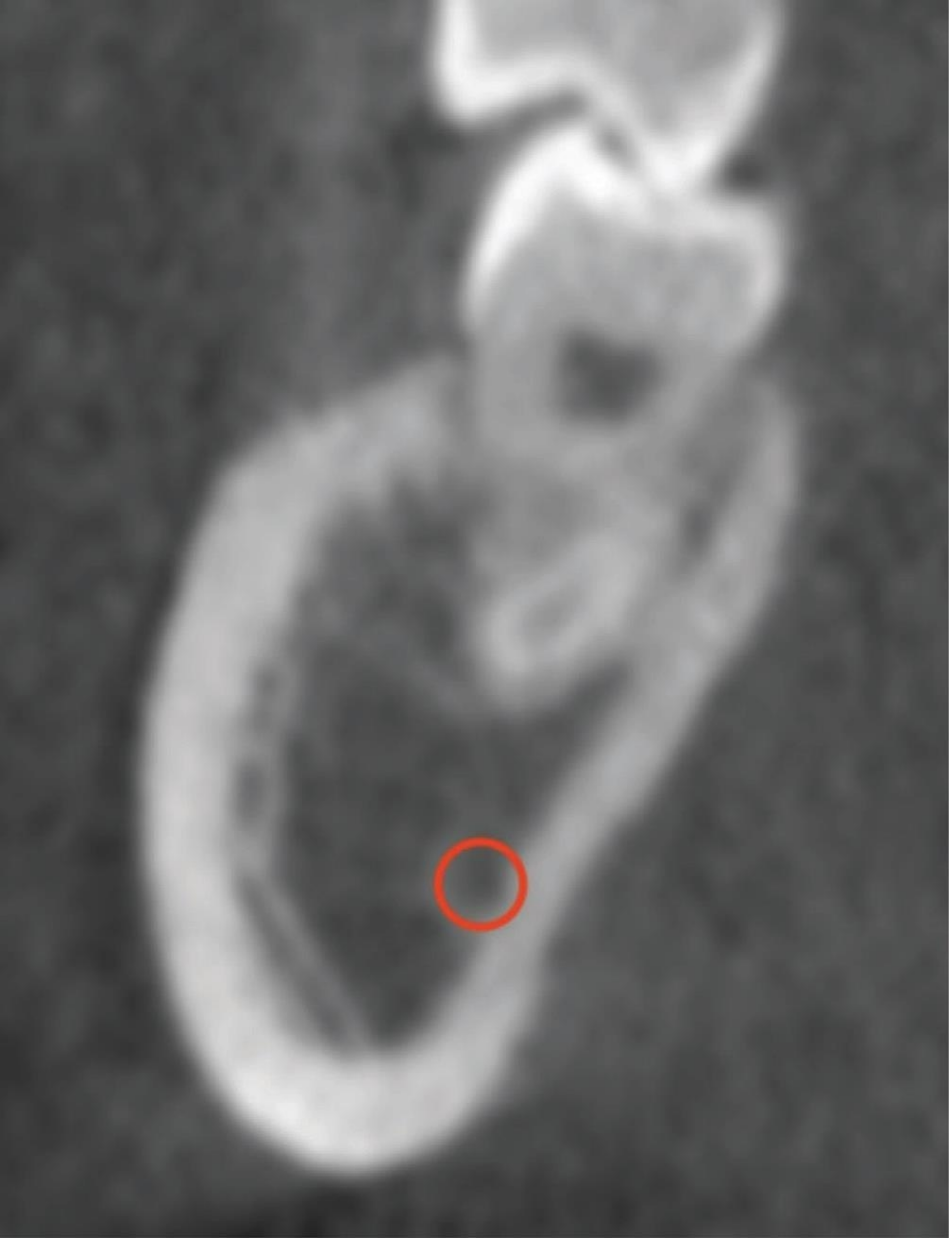
Ridge morphology exhibiting an increased risk of lingual cortical plate perforation during implant therapy with existing classification systems.

Periapical and panoramic radiographs have been widely used to assess potential implant sites. However, they provide a two‐dimensional view of the anatomical structures; it is not possible to determine the buccolingual width of the alveolar process [14]. To overcome this shortcoming, clinicians have used bone‐sounding/mapping and palpation techniques to estimate the buccolingual bone width. However, these methods cannot provide accurate details of bone morphology and anatomical variations. Additionally, the buccolingual relationship between the IAC and the implant site could not be determined. Cone‐beam computed tomography (CBCT) has become the imaging modality of choice for implant planning because it provides a three‐dimensional view of the region of interest, allowing clinicians to determine the relationship between the implant site and adjacent anatomical structures. Current classification systems are limited, particularly in their lack of integration of key anatomical parameters such as lingual concavity morphology and IAC position [15–17]. A clinical scenario where current classification systems cannot be applied is when the crestal alveolar bone is wide, lingual concavity is not prominent, the base is narrow, and there is exaggerated basal buccal tilt of the mandible. In such scenarios, there is an increased risk of lingual cortical plate perforation even though there is no lingual undercut (Figure 1).

The aim of the study was to revisit the posterior mandible morphology relevant to implant therapy with CBCT imaging [18]. Our objectives were to develop a novel classification system that determines the correlation between various anatomical landmarks of the posterior mandible, incorporating ridge shape, undercut characteristics, and canal location, to improve preoperative risk assessment and implant planning accuracy.

## 2. Materials and Methods

This retrospective study was conducted at the Dental Hospital, Dubai Health, Dubai, UAE, in accordance with the 1975 Declaration of Helsinki. Informed consent was obtained from the patients included in the study. The ethical approval for the study (MBRU IRB‐2021‐52) was obtained from the Institutional Review Board of the Mohammad Bin Rashid University of Medicine and Health Sciences, Dubai, UAE. One hundred ninety‐five CBCT volumes, out of a total of 350, were included in the study, as per the patient selection criteria stated below. The CBCT scans were acquired using a Sirona Galileos (Sirona Dental Systems GmbH, Bensheim, Germany) with a standard imaging protocol: 15 × 15 × 15 cm^3^ FOV, 0.15 mm voxel size, 85 kVp, 28 mA, and 14.21 s. The scans were de‐identified before analysis.

The Cochrane sample size for simple random sampling is given by the formula:

n=Zα/22p1−pd2,

where *p* is the proportion of lingual concavity, *d* is the precision of the estimate, and $z_{\alpha /2}^{2}$ is the quantile of the 95% confidence interval.

Considering a relative precision of 25% and assuming a maximum permissible limit of 25% for *p*, and an estimated addiction proportion of lingual concavity of 23%, then, the calculated precision will be (25/100) × 23 = 0.25 × 23 = 5.75. This means that we will be able to detect a “*p*” (proportion) of 17.25% or more (half the value of relative precision on either side of “*p*”–> ± 5%: 35%–28.75%). We wanted to estimate the proportion “*p*” of lingual concavity within 5% points with 95% probability, and therefore, ignore the stratification and two‐stage design of the sample and assumed the simple random sampling formula above. If the maximum value of “*p*” is to be 23%, then, the formula gives the value of *n* as we have used Type III; therefore, the sample size was 1.96 × 1.96 × 0.15 × (1–0.15)/(0.05 × 0.05) = 195.

The patients included in the study were above 19 years of age, with no restrictions on gender or ethnicity, had preoperative CBCT scans, at least one missing mandibular first molar, adequate bone height to accommodate an 8 mm long dental implant, and a crestal bone width of at least 3.5 mm. Patients receiving bone grafts with implant therapy, pathosis in the area of interest, congenital and developmental disorders, and abnormal ridge morphology due to trauma were excluded.

The primary investigator (FA) analyzed the CBCT volumes using OsiriX MD (Pixmeo SARL, Geneva, Switzerland) on two daisy‐chained Dell UP3017 monitors (Dell Corporation, Round Rock, Texas, USA) connected to a MacBook Pro (Apple Inc., Cupertino, California, USA) in a room with controlled ambient lighting. The 3D MPR (Multiplanar Reconstruction) image analysis tool was used to analyze the posterior mandible.

**Figure 2:**
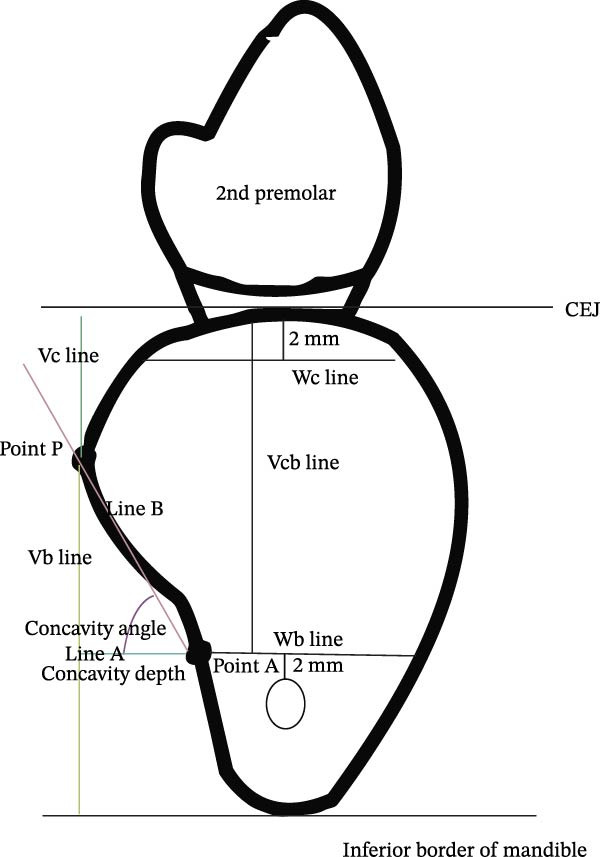
Illustration of the methodology used for determining the linear and angular measurements of the posterior mandible in the CBCT volumes. Line A (green, horizontal line); Point A (intersection of Line A and lingual cortical plate); Point P (most prominent point on lingual cortical plate); Wc (width of the alveolar ridge at crest); Wb (width of the alveolar ridge at base); Vcb (alveolar ridge height); Vb (alveolar ridge height below Point P); Vc (alveolar ridge height above Point P; Lingual concavity depth (distance between Point and Line Vb); Lingual concavity angle (formed by connecting Point P, Point A, and Line A).

The morphological measurement and assessment methodology were adapted from Chan et al. [19]. If the second molar was present, a cross‐sectional image through the midpoint of the first molar edentulous ridge site was chosen. If the second molar was absent, a cross‐sectional image was selected at a point 5 mm distal to the second premolar’s cementoenamel junction (CEJ). If both the second molar and second premolar were missing, a cross‐sectional image 13 mm distal to the distal border of the mental foramen was chosen (8 mm [average width of the 2^nd^ premolar] + 5 mm [half the width of 1^st^ molar]). Once the cross‐sectional image was selected, the IAC was identified, and a horizontal line, traversing the lingual and buccal cortical plates, was drawn 2 mm coronal to the superior border of the IAC (Line A). The intersection of Line A and the lingual cortical plate was labeled as Point A. The most prominent point on the lingual cortical plate was labeled as Point P. The buccolingual dimension 2 mm apical to the alveolar crest was chosen for horizontal Line Wc and 2 mm coronal to the superior border of the IAC for horizontal Line Wb (Line Wb is part of Line A). Three linear distances were then measured: from the alveolar ridge crest to Line Wb (Line Vcb), from Point P to the inferior border of the mandible (Line Vb), and from the CEJ to Point P (Line Vc). The depth of the lingual concavity was determined by measuring the distance between Point and Line Vb. The angle formed by connecting Point P, Point A, and Line A was denoted as the concavity angle (Figure 2).

### 2.1. Statistical Analyses

IBM SPSS Statistics for Windows version 29.0 (SPSS Inc., Chicago, IL, USA) was used for data analysis. Categorical variables were described using proportions, and continuous variables were described by measuring tendency and dispersion. Categorical variables were cross‐tabulated to examine the independence between variables; the *χ* ^2^‐square test, or Fisher’s exact test, as appropriate, was used for such variables. The Kolmogorov–Smirnov test was used to assess the normality of continuous variables. The Mann–Whitney test was used to compare the means of two groups, whereas the Kruskal‐Walli’s test was used to compare the means of three or more groups. If the data fulfilled the condition of normality, *t*‐tests and ANOVA were used. The association between two continuous variables, which were normally distributed, was tested using the Pearson correlation coefficient. The Spearman correlation coefficient was used for non‐normal continuous data. The intraobserver agreement was assessed using Kappa analysis, demonstrating consistency in the investigator’s analysis of the CBCT volumes. A *p*‐value <0.05 was considered significant in all statistical analyses.

The STROBE cross‐sectional checklist was used for preparing the manuscript.

## 3. Results

### 3.1. Study Population and Ridge‐Type Distribution

The study included a sample of males and females distributed across six age groups (Table 1). First molars were present in approximately two‐thirds of cases on both sides. Across the cohort, the undercut (U) ridge type predominated bilaterally, followed by the parallel (P) type, whereas the convergent (C) type was least frequent (Table 1). This distribution pattern was consistent between the left and right mandible, indicating a symmetrical prevalence of ridge morphology.

**Table 1:** Sample characteristics and alveolar ridge types.

Items	Number (%)
Demographics	
Gender	
Male	90 (46.2)
Female	105 (53.8)
Age	
20–29	40 (20.5)
30–39	68 (34.9)
40–49	38 (19.5)
50–59	31 (15.9)
60–69	15 (7.7)
70±	3 (1.5)
Clinical and imaging characteristics	
Left: First molar present	122 (62.6)
Right: First molar present	120 (61.5)
Left: Ridge type	
C	36 (18.5)
P	53 (27.2)
U	106 (54.4)
Right: Ridge type	
C	28 (14.4)
P	65 (33.5)
U	101 (52.1)

Abbreviations: C, convergent; P, parallel; U, undercut.

### 3.2. Primary Morphometric Differences by Sex

Sex‐related differences were observed in ridge dimensions. Males consistently exhibited greater alveolar ridge height and greater ridge height above the P point on both sides compared with females (Table 2). These differences reached statistical significance and were directionally consistent, suggesting a systematic sexual dimorphism in vertical ridge dimensions rather than isolated findings.

**Table 2:** Morphological measurements and their statistical significance by gender, age, presence/absence of first molar, and presence/absence of lingual undercut.

Gender	Left			Right		
	Male	Female	*p*‐Value	Male	Female	*p*‐Value
Width of alveolar bone at crest	9.28 (2.87)	8.57 (2.45)	0.068	9.16 (2.49)	8.6 (32)	0.095
Width of alveolar bone at base	11.22 (2.08)	10.69 (2.21)	0.092	11.13 (1.76)	10.67 (1.98)	0.091
Alveolar ridge height	**15.23 (3.73)**	**13.4 (3.46)**	**<0.001**	**15.49 (3.94)**	**13.66 (3.33)**	**0.047**
Ridge height above P point	**18.47 (4.62)**	**16.02 (4.6)**	**<0.001**	**17.98 (4.32)**	**16.77 (4.98)**	**<0.001**
Ridge height below P point	10.16 (5.01)	9.76 (5.52)	0.370	**10.19 (4.62)**	**9.2 (5.59)**	**0.044**
Lingual concavity depth	1.72 (1.46)	1.56 (1.32)	0.521	1.93 (1.56)	1.7 (1.48)	0.436
Lingual concavity angle	57.35 (28.72)	55.24 (28.65)	0.259	57.38 (27.6)	52.75 (31.45)	0.636

**Age**	**<40 years**	**≥40 years**		**<40 years**	**≥40 years**	

Width of alveolar bone at crest	**9.29 (2.53)**	**8.41 (2.77)**	**0.023**	**9.35 (2.24)**	**8.25 (2.48)**	**<0.001**
Width of alveolar bone at base	11.13 (1.9)	10.69 (2.44)	0.154	10.95 (1.95)	10.8 (1.83)	0.566
Alveolar ridge height	**14.79 (3.28)**	**13.57 (4.06)**	**0.025**	**15.16 (3.18)**	**13.69 (4.19)**	**0.008**
Ridge height above P point	17.06 (4.71)	17.26 (4.84)	0.774	17.29 (4.84)	17.39 (4.48)	0.887
Ridge height below P point	10.01 (5.04)	9.86 (5.59)	0.610	9.87 (5.17)	9.4 (5.21)	0.433
Lingual concavity depth	1.64 (1.36)	1.62 (1.41)	0.889	1.85 (1.65)	1.76 (1.34)	0.853
Lingual concavity angle	57.09 (28.41)	55.13 (29.02)	0.508	54.18 (30.8)	55.77 (28.28)	0.985

**First molar**	**Yes**	**No**		**Yes**	**No**	

Width of alveolar bone at crest	**10.04 (1.92)**	**6.99 (2.66)**	**<0.001**	**10.14 (1.65)**	**6.81 (1.99)**	**<0.001**
Width of alveolar bone at base	11.07 (2.06)	10.71 (2.32)	0.134	10.9 (2.04)	10.84 (1.64)	0.834
Alveolar ridge height	**15.72 (2.94)**	**11.78 (3.51)**	**<0.001**	**15.64 (3.28)**	**12.69 (3.71)**	**<0.001**
Ridge height above P point	**18.11 (4.79)**	**15.55 (4.30)**	**<0.001**	**18.73 (4.82)**	**15.14 (3.45)**	**<0.001**
Ridge height below P point	**10.53 (5.49)**	**8.97 (4.79)**	**0.043**	**9.48 (5.48)**	**9.95 (4.68)**	**<0.001**
Lingual concavity depth	1.75 (1.44)	1.44 (1.27)	0.087	**2.11 (1.61)**	**1.33 (1.22)**	**<0.001**
Lingual concavity angle	56.92 (29.3)	55.04 (27.62)	0.157	**58.64 (28.3)**	**48.89 (31.2)**	**0.005**

**Lingual undercut**	**With undercut**	**Without undercut**	***p*-Value**	**With undercut**	**Without undercut**	***p*-Value**

Width of alveolar bone at crest	8.90 (2.74)	8.84 (2.05)	0.465	8.92 (2.43)	8.82 (2.13)	0.615
Width of alveolar bone at base	10.98 (2.17)	10.33 (1.98)	0.121	8.92 (2.43)	10.58 (1.83)	0.366
Alveolar ridge height	14.16 (3.75)	15.31 (2.85)	0.123	**14.32 (3.68)**	**16.35 (3.27)**	**<0.001**
Ridge height above P point	**17.53 (4.39)**	**12.59 (6.56)**	**0.006**	**17.78 (3.68)**	**15.17 (5.5)**	**0.006**
Ridge height below P point	**9.46 (4.72)**	**15.78 (7.87)**	**0.002**	**9.08 (4.74)**	**13.55 (6.37)**	**0.026**
Lingual concavity depth	**1.72 (1.4)**	**0.61 (0.64)**	**0.001**	**2.01 (1.55)**	**0.71 (0.69)**	**<0.001**
Lingual concavity angle	57.67 (27.24)	38.82 (39.95)	0.152	56.60 (27.4)	46 (39.71)	0.902

*Note:* Statistically significant values are in bold.

### 3.3. Age‐Related Differences

When stratified by age, individuals younger than 40 years demonstrated greater alveolar crest width and ridge height bilaterally compared with those aged 40 years and older (Table 2). Although statistically significant, these differences followed a modest gradient, indicating a gradual reduction in ridge dimensions with increasing age rather than an abrupt change.

### 3.4. Influence of First Molar Presence

The presence of the first molar was associated with substantially greater ridge width and height on both sides of the mandible (Table 2). This effect was most pronounced for crest width and total ridge height, while measurements above and below the P point showed a similar directional trend. On the right side, molar presence was additionally associated with greater concavity depth and larger concavity angle, reflecting a more pronounced lingual contour in dentate sites. Collectively, these findings suggest that first molar presence is a strong modifier of alveolar ridge morphology.

### 3.5. Lingual Undercut and Ridge Morphology

Sites exhibiting a lingual undercut showed distinct vertical and concavity‐related characteristics compared with non‐undercut sites. On both sides, undercut presence was associated with greater ridge height above the P point and increased concavity depth, whereas ridge height below the P point tended to be reduced (Table 2). These opposing vertical patterns indicate that undercuts are primarily driven by superior ridge contour rather than basal bone height.

### 3.6. Ridge‐Type Specific Morphometric Patterns

Clear and systematic morphometric differences were observed among ridge types (Table 3). C‐type ridges were generally narrower and lower in height, whereas P‐type ridges showed intermediate dimensions across most parameters. U‐type ridges displayed the greatest ridge height above the P point and the deepest concavities, particularly on the right side, consistent with their defining morphology. These patterns were statistically significant and consistent on both sides, supporting the internal validity of the ridge classification system.

**Table 3:** Comparison of morphological measurements by ridge type.

Measurements	Left		Right	
	Mean (SD)	*p*‐Value	Mean (SD)	*p*‐Value
Width of the alveolar bone at crest				
C	**6.75 (3.19)**	**<0.001**	**6.66 (2.21)**	**<0.001**
P	**9.44 (2.24)**		**8.95 (2.17)**	
U	**9.35 (2.32)**		**9.4 (2.29)**	
Width of the alveolar bone at base				
C	**10.69 (1.99)**	**0.046**	10.87 (1.15)	0.328
P	**10.42 (1.98)**		10.60 (1.85)	
U	**11.28 (2.26)**		11.05 (2.07)	
Alveolar ridge height				
C	**12.59 (3.12)**	**0.001**	**12.64 (3.82)**	**0.002**
P	**15.43 (3.01)**		**15.54 (3.61)**	
U	**14.22 (3.98)**		**14.3 (3.56)**	
Ridge height above the P point				
C	**14.81 (3.92)**	**0.002**	**14.8 (3.53)**	**0.007**
P	**17.04 (5.51)**		**17.49 (5.15)**	
U	**18.0 (4.36)**		**17.91 (4.44)**	
Ridge height below the P point				
C	10.38 (5.42)	0.173	10.39 (5.16)	0.141
P	11.23 (6.49)		10.5 (5.75)	
U	9.15 (4.40)		8.96 (4.73)	
Lingual concavity depth				
C	**1.19 (0.96)**	**0.021**	**1.16 (1.09)**	**<0.001**
P	**1.42 (1.22)**		**1.37 (1.03)**	
U	**1.89 (1.53)**		**2.27 (1.74)**	
Lingual concavity angle				
C	52.68 (29.99)	0.207	**43.42 (31.63)**	**<0.001**
P	57.42 (31.45)		**59 (32.33)**	
U	56.82 (26.82)		**55.97 (26.5)**	

*Note:* Statistically significant values are in bold.

Abbreviations: C, convergent; P, parallel; U, undercut.

### 3.7. Association Between Ridge Type and Lingual Undercut

A strong association was observed between ridge type and the presence of a lingual undercut. Nearly all U‐type ridges demonstrated an undercut on both sides, whereas C‐type and P‐type ridges showed undercuts less consistently (Table 3). This graded relationship supports the clinical relevance of ridge‐type classification, as morphology reliably reflects the likelihood of undercut presence.

## 4. Discussion

The posterior region of the mandible and floor of the mouth contain vital structures such as the inferior alveolar nerve and vessels, lingual nerve, submandibular salivary gland, and lymph nodes. Injury to any of these structures, for example, by lingual cortical plate perforation during implant placement, may lead to complications, which can sometimes be fatal [6, 19]. A thorough assessment of the anatomical structures of the proposed implant site before implant placement can reduce or eliminate such complications. An in‐depth knowledge of mandibular morphology and variations can help formulate an optimal treatment plan.

The most prevalent ridge type in our study was the U‐type (54.4% on the left and 52.1% on the right), followed by the P‐type (27.2% on the left and 33.5% on the right) and the C‐type (18.5% on the left and 14.4% on the right). These findings align with those of Chan et al. [19], Herranz‐Aparicio et al. [20], and Yoon et al. [21], who employed similar methods, classification systems, and landmarks for ridge‐type determination. On the contrary, Salemi et al. [22] found that the C‐type ridge was more prevalent than the P‐type, although the U‐type ridge was still the most prevalent. Although the study employed a similar classification method, only patients with missing first molars were included, which may explain the difference in the findings. Watanabe et al. [4] found the C‐type ridge (round ridge) to be the most prevalent, followed by A‐type (lingual concavity) and B‐type (buccal concavity) as the least prevalent. These findings differ from our study, which could be due to the use of a different classification method and the study cohort’s predominantly Japanese ethnicity, compared to our study, which had no restrictions on ethnicity. A correlation was found between gender and the height of the alveolar ridge, as well as the height of the alveolar ridge above the P point on both the left and right sides, with higher values observed in males. The height of the alveolar ridge below the P point showed a correlation only on the right side. These findings align with those of Sameli et al. [22] and Watanabe et al. [4], indicating that males have higher and broader alveolar ridges, which may facilitate a relatively safer implant placement procedure. Chan et al. [19] found a correlation between gender and height of the alveolar ridge below the P point only, while Kamburoglu et al. [23] and Herranz‐Aparicio et al. [20] found no correlation between the height of the alveolar ridge and gender.

Our study found no correlation between gender and the width of the alveolar bone at the crest or base. Chan et al. [19] showed similar findings concerning the width of the alveolar bone at the base; however, a correlation was found between the width of the alveolar bone at the crest and gender. On the contrary, Herranz‐Aparicio et al. [20] did not find a correlation between the width of the alveolar bone at the crest and gender, while a correlation was found between the width at the base and gender. Furthermore, Salemi et al. [22] found a correlation between gender and the width at the crest and base. The disagreement in the findings could be due to the patient selection criteria; only patients with missing first molars were included.

We found a correlation between the height of the alveolar ridge, the width of the alveolar bone at the crest, and age, with those below 40 years of age showing higher values. This finding suggests that individuals under 40 have higher and broader alveolar ridges at the crest, which translates to a relatively safer implant placement procedure. Our findings align with that of De Souza et al. [24]; however, Herranz‐Aparicio et al. [20] found a negative correlation between the width at the crest and age, as well as no correlation between the height of the ridge and age.

A positive correlation was found between the height of the alveolar ridge, height above and below the P point, the width of the alveolar bone at the crest, age, and the presence of first molars, where patients with first molars showed higher values. This indicates that people with their first molars present have a higher alveolar ridge that is broader at the crest, facilitating a safer implant placement procedure. To our knowledge, no previous studies have compared these parameters.

We found a correlation between the height of the alveolar bone below and above the P point, concavity depth, and the presence of an undercut. Patients with an undercut showed a higher alveolar ridge above the P point, a deeper concavity, but a lower value of the height of the bone below the P point. These findings suggest that individuals with an undercut may be at a higher risk of lingual cortical plate perforation due to the deeper concavity. To our knowledge, no previous studies have compared these parameters.

A correlation was found between the height of the alveolar bone, height above and below the P point, the width of the alveolar bone at the crest, the width of the bone at the base, and ridge type on the left and right sides, except for bone width at the base and ridge type on the right. The findings varied among different ridge types, with the C‐type exhibiting the lowest values and the P‐ and U‐types being close to each other; the P‐type showed higher values only at the height of the alveolar bone. Knowing the ridge type is beneficial because it suggests the need for further assessment and a modified procedure; for example, the C‐type ridge will have a higher chance of needing bone augmentation due to its narrow width at the crest. To our knowledge, no previous study has compared these parameters.

No correlation was found between the depth and angulation of concavity and gender. The finding is in agreement with Rajput et al. [8], Herranz‐Aparicio et al. [20], Yoon et al. [21], Salemi et al. [22], Chan et al. [19], and Parnia et al. [25]. However, Ramaswamy et al. [17] showed a correlation between gender and concavity depth, with males having deeper concavities. No correlation was found between the depth and angulation of the concavity and age. The finding is in agreement with Salemi et al. [22], Chan et al. [19], and Parnia et al. [25]. However, Herranz‐Aparicio et al. [20] and Kamburoglu et al. [23] found a negative correlation between concavity depth and age, while Yoon et al. [21] found a negative correlation between concavity height and age.

We found a correlation between the lingual concavity depth and the presence of the first molar, with the concavity being deeper in those with their first molars present. This finding aligns with those of Uchida et al. [7] and Kamburoglu et al. [23].

Our study found a correlation between the lingual concavity depth and the presence of an undercut, the concavity being deeper in those with an undercut. To our knowledge, this is the only study that has compared these parameters. However, Braut et al. [26] classified the undercut as either influential or non‐influential and found it to be more influential in the molar region.

Jung [27] classified the mandibular morphology into Type I (concavity depth of less than 2 mm), Type Ⅱ (concavity depth between 2 and 3 mm), and Type Ⅲ (concavity depth of more than 3 mm). Although it provides sufficient information about the depth and angulation of the concavity, it lacks information about the ridge shape, which limits its use due to a lack of details about the height and width of the ridge.

Watanabe et al. [4] classified the alveolar ridge into Type A, rounded buccally and concave lingually; Type B, rounded lingually and concave buccally; Type C, rounded on both sides. This classification provides adequate information about the ridge shape but none about the depth of concavity. The study’s methodology did not account for the width of the alveolar ridge at the crest, which may indicate a need for bone augmentation prior to implant placement.

Braut et al. [26] classified the undercut as absent, present influential, that is, located in the path of implant placement, and present noninfluential, that is, present without interfering with the path of implant placement. This classification is useful in minimizing the risk of lingual cortical plate perforation, but does not provide any information about the ridge shape and form.

Chan et al. [19] classified the mandibular ridge morphology into convex (C), parallel (P), and undercut (U)‐types based on the shape of the alveolar ridge and the presence of lingual concavity. A ridge with a narrow base, wide crest, and prominent P point creating an undercut was classified as U‐type. A ridge with a base wider than the crest and no undercut was classified as C‐type, and with roughly parallel cortical plates and no undercut as P‐type. Many studies have employed this classification because it provides information about the shape of the alveolar ridge and the presence or absence of a lingual undercut; however, the influence of the undercut on implant placement remains undetermined. In addition, all ridge types with influential undercuts, including the P‐ and C‐types, were grouped as U‐type. To overcome these limitations, we have introduced a new term, D‐type, as part of the new classification system. The D‐type ridge is narrow at its base, wide at its crest, and has an undercut, which can be either influential or noninfluential.

### 4.1. The CPDU Classification System

The new classification system aims to mitigate the limitations of existing systems. Considering the shape of the alveolar ridge, lingual concavity depth and angle, and the position of the IAC, this provides thorough information about the parameters essential for successful implant therapy in the posterior mandible.

**Figure 3:**
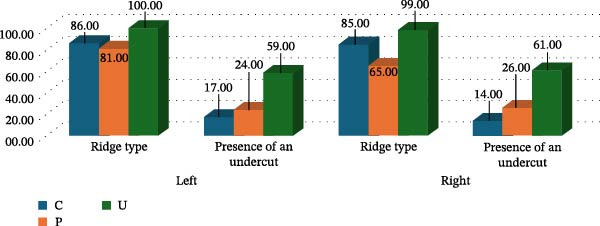
Correlation between ridge type and undercut presence. All ridge types exhibit undercuts.

**Figure 4:**
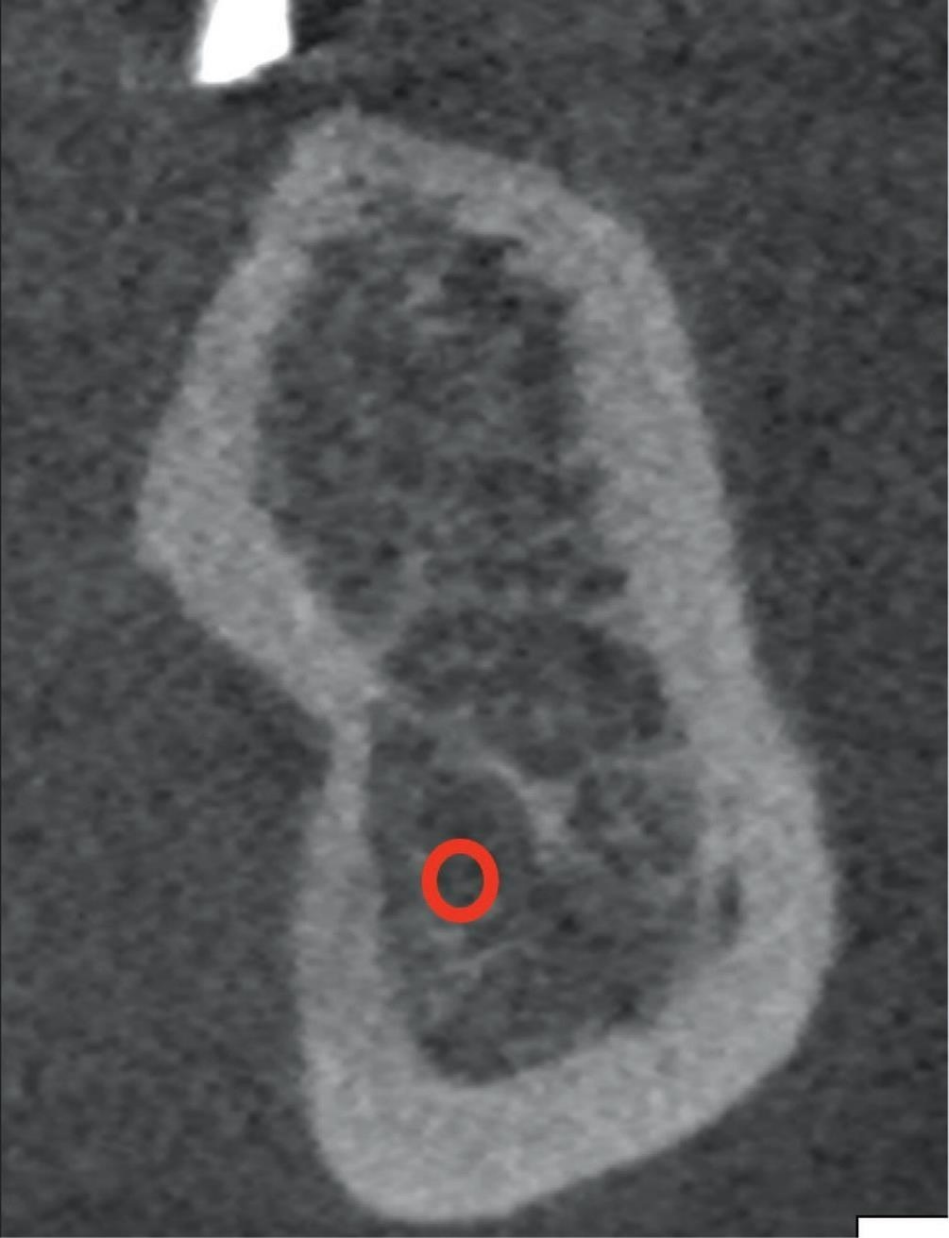
P–U–I type ridge.

**Figure 5:**
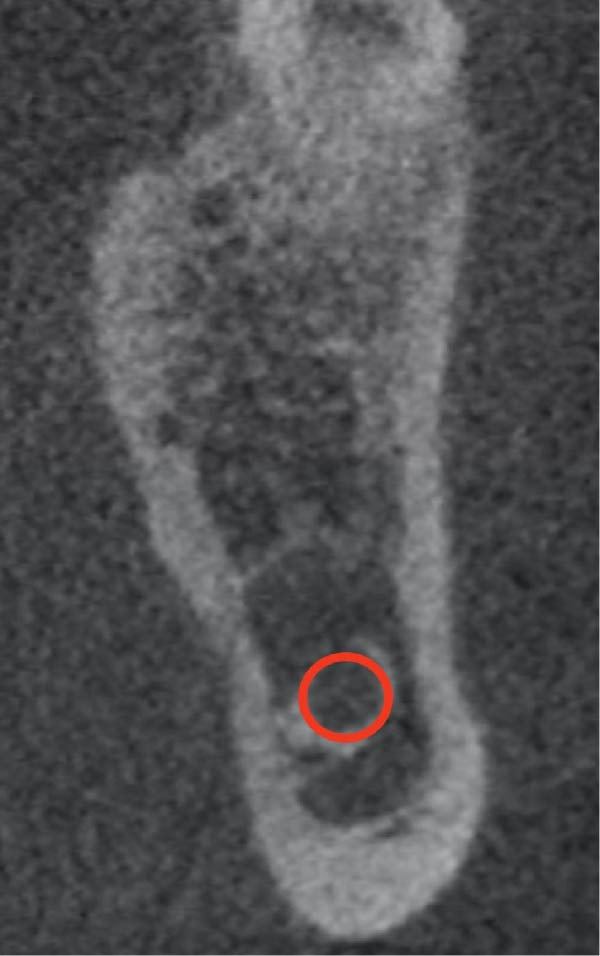
D–I type ridge.

**Figure 6:**
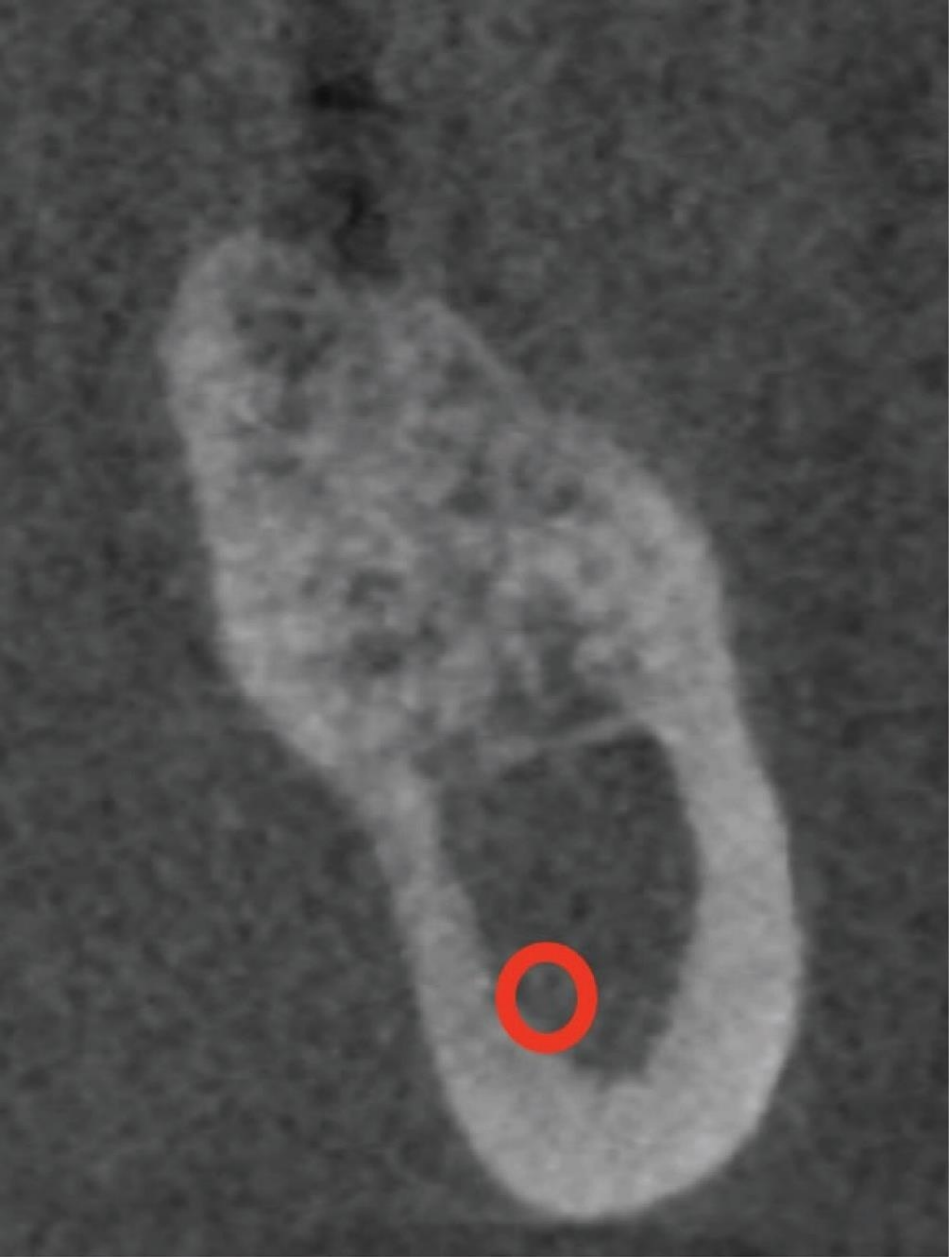
C–U–I type ridge.

We first categorized the alveolar ridge into three types based on shape: C (convergent)—wide at the base and narrow at the crest; P (parallel)—roughly the same width at the crest and base; D (divergent)—narrow at the base, wide at the crest, and has an undercut. Our study showed that lingual undercut (lingual concavity) can be present in any ridge type (Figure 3); therefore, if the undercut was present, the letter U was added after the letter representing the shape of the alveolar ridge. Step 2 does not apply to the D‐type ridge since an undercut is inherent to a D‐type ridge. If the undercut was absent in the C‐ and P‐type ridges, the letter representing the ridge shape sufficed. The third and final step was to determine whether the undercut was influential (I), that is, present above the IAC with a depth and angulation that may pose a risk of lingual cortical plate perforation or non‐influential (N); an appropriate letter was added to the classification accordingly. For example, a parallel ridge type with an influential undercut will be classified as P–U–I type (Figure 4), a divergent ridge with an influential undercut will be denoted as D–I type (Figure 5), and a convergent ridge with an influential undercut will be termed as C–U–I type (Figure 6). An alveolar ridge classified as D–I type conveys that it is narrow at the base, wide at the crest, and has an undercut above the IAC, posing lingual cortical plate perforation risk during implant therapy.

## 5. Conclusion

This study introduces and validates a new posterior mandible alveolar ridge classification system, which is a morphology‐based framework designed to enhance preoperative assessment for implant therapy. By categorizing ridge types into C (convergent), P (parallel), D (divergent), and U (undercut), the system provides a structured approach to evaluating anatomical variations that influence implant planning. The identification of D‐type ridges, characterized by a buccolingual divergence and proximity to the IAC, has significant implications for surgical decision‐making. We recommend:

• Narrow‐diameter or short implants in D‐type ridges to reduce the risk of canal encroachment.
• Angled drilling techniques or guided surgery to optimize implant trajectory and avoid neurovascular compromise.
• Preoperative and high‐resolution CBCT imaging with appropriate voxel size and field‐of‐view to accurately assess ridge morphology and canal positioning.
• Considering D‐type ridges as higher‐risk zones requiring enhanced planning and possibly staged procedures.

By integrating the new classification system into routine diagnostic workflows, clinicians can improve safety, precision, and long‐term success of implant therapy. Future studies should explore multicenter validation and correlate ridge types with clinical success rates to further establish the utility of the CPDU classification system.

## Author Contributions

Conceptualization, supervision: Moosa Abdulla Abuzayeda. Methodology, investigation: Faisal Alqaood, Jahanzeb Chaudhry, and Moosa Abdulla Abuzayeda. Data curation: Faisal Alqaood. Formal analysis: Amar Hassan Khamis and Faisal Alqaood. Writing – original draft: Faisal Alqaood, Jahanzeb Chaudhry, Amar Hassan Khamis, Keyvan Moharamzadeh, and Moosa Abdulla Abuzayeda.

## Funding

The authors have nothing to report.

## Conflicts of Interest

The authors declare no conflicts of interest.

## Data Availability

The data that support the findings of this study are available upon request from the corresponding author. The data are not publicly available due to privacy or ethical restrictions.
